# Garments and Footwear for Chronic Pain

**DOI:** 10.3389/fpain.2021.757240

**Published:** 2021-10-25

**Authors:** Maral Tajerian, Jaqueline Garcia

**Affiliations:** ^1^Department of Biology, Queens College, City University of New York, New York, NY, United States; ^2^The Graduate Center, City University of New York, New York, NY, United States

**Keywords:** garment, footwear, wearables, chronic pain, adaptive clothing, hypersensitivity

## Abstract

In most human societies, wearing clothing and shoes, particularly in public settings, is commonplace and may even be legally required. Consequently, there is an abundance of clothing and footwear options for individuals of different ages, genders, body shapes and catering to different needs such as workwear and active-wear. However, many of the available options may not be viable for the millions and pain sufferers worldwide, indicating a need for adaptive apparel for the pain patient. In this perspective manuscript, we focus on the availability and efficacy of clothing designed to prevent pain in the general population as well as reduce or treat pain in pain patients. Furthermore, we put forth some considerations for the construction of adaptive garments. Such efforts and needed and could significantly improve well-being and quality of life in the pain patient.

## Introduction

Perhaps with the exception of the hermit crab, humans are the only organisms that seek to actively clothe themselves as a barrier against the elements as well as tools of expression of cultural, socioeconomic, and gender identity. Indeed, clothing and footwear are the necessary evils of modern society where the expectation of being clad in the appropriate attire is a reasonable assumption. This is particularly evident for those working outside the home or in a social setting, where specific dress codes could be put in place due to social or occupational needs, including appropriate workwear for particular tasks. While most people have sufficient access to the required apparel, many chronic patients are limited in their choice of attire due to sensory abnormalities (tactile/heat/cold allodynia or hyperalgesia), limited range of movement (joint pain), poor fit (swollen limbs), etc. For instance, putting on a pair of trousers may not be given much thought by a healthy individual, but may prove to be extremely challenging for a patient with rheumatoid arthritis to fasten the buttons, for a back pain patient to bend over enough to get the legs through, or for a fibromyalgia patient to tolerate the mechanical excitation that the garment may provoke.

In general, useful apparel must satisfy many conditions, including some that are purely ornamental (spikes on jackets, very high heels, embroidered designs, etc.). The item usually has to be aesthetically pleasing, contemporary, comfortable, economically feasible, and indicative of culture and gender identity. It is therefore unsurprising that the design and production of garments is often subject to trial and error and could potentially marginalize, or at least underserve, select populations including those with various medical conditions.

This perspective targets a wide audience of pain patients, clinicians, basic scientists, as well as garment designers and producers. It summarizes published findings on the use and efficacy of adaptive apparel and footwear both for the prevention and treatment of painful conditions. It also provides insight into the predicted future of adaptive garments for pain. Despite the rising popularity of smart fabrics and biofeedback, the current perspective will exclude wearable items that serve monitoring purposes since there are only very few reports on the subject (1).

## Prevention of Clothing or Footwear-Induced Pain in the General Population

Clothing and footwear, in addition to serving pragmatic uses, are signifiers of cultural identity, gender and sexual identity, socioeconomic status, etc. As such, the need to maximize comfort and minimize pain is self-evident. Furthermore, failure to do so may result in injury and chronic pain. Most comfortable wear is focused on age (both infants and the elderly), as well as occupational needs. Assistive dressing devices including adaptive garments have been increasing in demand and will continue to do so with the rising elderly population (2, 3). Popular assistive dressing devices include shoehorns, sock aides, button aids and altered household products such as the dressing stick, which is a modified broom handle (3).

### Garments

Evaluation of garment construction (design and materials) has been used to investigate the comfort of garments with a focus on improving fit. In pain-free subjects, friction from clothing items as well as ill-fit can cause discomfort and skin sensitivity (4). Improvements to garment comfort and the use of specialized garments could be required. The sports bra, for example, has been shown to help relieve symptoms of mastalgia (5). In a population of British army recruits, professional fittings for sports bras resulted in a decrease in reported breast pain (6). However, some discomfort remained in those professionally fitted, where 32% reported sizing issues and 70% reported rubbing/ chafing, indicating a need for a more personalized approach. Indeed, ideal sports bras vary depending on breast size, age and activity type (7–10). Bra strap placement is important to consider as it can affect range of motion and pain threshold. For example, despite the widespread use of parallel straps, crossed straps were found to be associated with a wider range of motion and decreased discomfort (11). Many sports activities use specialized apparel both for aesthetic and practical reasons. The use of a posture-cueing, compressive shirt was linked with self-reported decrease in spine discomfort and increase in post-ride recovery (12).

The work environment brings up clothing challenges based on the job requirements, particularly where carrying heavy loads is involved. Specialized garments to prevent back pain while carrying heavy loads date back to centuries and are present across different cultures. The Nepalese Patuka is a garment worn around the waist to aid in load bearing and has shown some modest efficacy in preventing back pain in mountain porters (13). More recently, the use of a load-bearing vest and thigh holster resulted in less discomfort in the Swedish police (14) and in a randomized crossover study, exosuits showed favorable results of pain reduction compared to the control conditions in surgeons (15). Further study of comfortable and well-fitting garments will help resolve both person-specific and environment-specific garment challenges.

### Footwear

One of the objectives of pain reduction in footwear is to avoid ill-fit. This is complicated by the poor operationalization of ill-fitting footwear whose definitions focus on the absence of comfort but lack consensus on specific criteria. Ill-fitting shoes have been shown to affect gait, lead to foot and ankle injuries such as skin lesions, sprains, or structural disorders or deformities and pain (16, 17). Among older populations, ill-fitting areas in shoes have been observed to be the toes, metatarsophalangeal joints and the foot sole (18). Creating custom therapeutic footwear can be time consuming and costly. Fortunately, personalized interventions are not the only solution. For those who stand for long periods of time during work, it has been observed that cushioned materials and insoles help with fatigue and discomfort (19). The use of mass produced commercially available shoes chosen for specific qualities may also help reduce pain. Plantar fasciitis treated with physical therapy benefitted from the use of shoes with an ultra-flexible midsole (20).

Well-fitting shoe criteria has come to include foot dimensions but as with ill-fitting footwear, there is a lack of consensus in the literature as to which dimensions should be prioritized. The inclusion of one dimension to define well-fitting shoes does not ensure proper fitting nor does it ensure the absence of reported discomfort. This can be seen in the case of coal miners who reported pain, calluses and discomfort despite wearing shoes with the correct length (21). Dobson et al. investigated the association of dimensions in coal miners' boots with subjective measures of fit, comfort, foot problems, lower limb pain and lower back pain. Heel and ball girth circumferences were, respectively found to significantly affect comfort ratings. Pain was significantly related to heel breadth, heel girth circumference, and instep height (21). Multifactorial analysis of foot dimensions could reveal significant clues about appropriate shoe designs and could result in clearer operational definitions of well-fitting and ill-fitting shoes.

## Adaptive/Therapeutic Garments for Chronic Pain

While the section above reviews the availability of garments that are designed to minimize pain or improve comfort for the general population, this section addresses the possibility of adaptive garments serving a therapeutic purpose for chronic pain patients. The interventions are often “alternative and complimentary” to pharmacological, behavioral, and surgical therapies and their precise mechanisms of action remain unknown. Below are some chronic pain conditions where adaptive clothing has been proposed or utilized. It is not surprising that the type of wearable, as well as its location of the body, is closely linked to the somatic manifestation of the pain condition.

### Fibromyalgia

Fibromyalgia syndrome is characterized by allodynia and hyperalgesia in various regions including superficial cutaneous tissues (22). Despite a few commercial ventures into developing adaptive clothing to accommodate such pain hypersensitivity, to our knowledge, there are no published studies that ascertain their widespread use or efficacy. There are, however, a few published reports on therapeutic interventions that employ the use of a wearable garment with the aim of ameliorating fibromyalgia symptoms and perhaps even guiding future clothing choices in patients. For instance, in a 60-day randomized controlled pilot study using bio-ceramic undershirts in 39 female fibromyalgia patients, the treatment group showed a significant reduction in pain as well as daily intake of analgesics (23). In a randomized trial of 50 patients, the use of wool undergarments and bedding for 6 weeks reduced pain levels and tender point counts (24).

### Back Pain

Compression garments are sometimes used in back pain patients and many interventions that are used as precautionary measures to prevent back pain can also be efficacious in its amelioration [for example, see (25)]. In a study of 112 patients, the use of corsets was linked with 60% compliance and excellent improvement in 37% of subjects (26). Low back pain experienced during pregnancy is sometimes improved by the use of maternity supportive garments [support belts (27) and shorts (28)] as evidenced by a systematic review of studies where the garments were the sole intervention used to treat back pain (29).

Most interventions for back pain focus on footwear rather than clothing with main emphasis on adapting the shoe insole to aid in pain relief. There is an abundance of foot orthoses and insoles that carry the promise of back pain abatement. Unfortunately, metanalyses of such studies often cite the lack of satisfactory evidence to support any claims of significant efficacy (30–32). More sophisticated devices may therefore be needed to be incorporated into footwear design, an approach that has been observed in recent years. For instance, in a double-blinded randomized controlled study, 83 back pain patients were given shoes equipped with electronic acupuncture. This device combines acupuncture and transcutaneous electrical nerve stimulation, both of which are already in clinical use. A 6-week treatment protocol indicated that the use of this therapeutic footwear had better treatment success than non-steroidal anti-inflammatory administration (33).

### Rheumatoid Arthritis

Most interventions for this patient population are focused on gloves and shoes/stockings and are implemented prior to resorting to surgical treatments. A review of seven clinical trials and one case study testing the efficacy of therapy gloves (thermal and/or compression) for a wide range of trial durations (1–52 weeks) demonstrated that the gloves improve symptoms such as such as pain, stiffness and swelling with marginal improvement in hand function (34). More recently, a systematic review of 4 randomized controlled trials evaluating the efficacy of compression gloves worn at night was inconclusive (35). Key characteristics for footwear targeted toward rheumatoid arthritis patients often include additional cushioning and a wide toe box. A systematic review and meta-analysis examining the effectiveness of therapeutic shoes in patients with rheumatoid arthritis concluded that the use of therapeutic footwear has a high likelihood of improving foot pain and foot function, while noting the need for additional definitive high-quality randomized controlled trials (36).

While promising, most of the abovementioned interventions are not established therapies. As pain awareness increases in the clinical, social, and commercial spheres, it is likely that we will observe an increase in scientific studies guiding the design and implementation of adaptive wear for the chronic pain patient.

## Considerations for Apparel Adapted to the Pain Patient

Apparel manufacture is a rapidly evolving business that responds to economical, societal, and technological changes.

### Design

Garment design incorporates multiple elements (proportion, color, shape, etc.) to produce a function and visually pleasing outcome. Design can influence factors such as moisture transfer, heat, range of movement, etc., all of which are important considerations for pain patients both during dressing/undressing and wearing the garment for a prolonged period of time. For instance, shirts with narrower silhouettes may be difficult to put on for patients with reduced range of motion in the shoulders, the microclimate provided by a tight sweater may be different from one that is more voluminous, appropriately sized sleeves may increase airflow to the body and reduce thermal load, and the availability of pockets may be a necessity for those with medical devices. Finally, the design should be aesthetically pleasing to ensure compliance by the wearer as well as improved psychological well-being.

### Materials

Fabric composition can influence its mechanical properties and thus the forces by which sensory terminals are stimulated in pain patients. In particular, fabric weight, rigidity, stretch, and recovery are important properties to consider and are often key factors in designing specialized garments such as sportswear and protective clothing. For adaptive garments for pain patients, it may be necessary to consider items comprising of fabric sections with different properties. For instance, fabrics with low friction may be utilized in areas with high tactile sensitivity such as the chest and neck area, while fabrics with more stretch could provide compression in the lower back area. Here, it's important to consider both fabric composition as well as the chemical finish applied to enhance garment performance. Anti-static finishes could be valuable in preventing sparks and shocks, particularly useful in patients who have to wear electronic medical devices. Phase-change finishes could minimize temperature extremes and provide a more comfortable environment for those with heat or cold hypersensitivity. Anti-stain finishes would make the garment easier to care for, thus making such garments a practical choice for individuals with physical disabilities.

Rapid advances in fabric engineering could inspire their mainstream use as daily apparel rather than localized therapies. For example, the use of anti-adhesive nanofibers sandwiching antimicrobial agents, analgesics, and human epidermal growth factor was shown to aid in postoperative pain relief and wound repair (37). Similar fibers could be used in specific parts of daily-wear garments for those patients who may benefit from topical analgesics. For pain patients suffering from chronic wounds, it is possible to envision the incorporation of one of many collagen-based hydrogels into socks, gloves, or even undergarments (38).

In addition to the fabric itself, care must be taken to optimize other components of garment construction such as fasteners. Buttons and zippers can be particularly challenging to those with joint pain, rigid snap buttons may be inaccessible for patients with myalgia, etc. As such, alternatives such as Velcro or magnetic fasteners may be employed.

Garment design and materials go hand-in-hand in creating comfortable and functional garments for pain patients. The availability of modeling and simulation techniques for garment design may facilitate the creation of adaptive garments. Attempts at improving fit and comfort of garments have come to incorporate the use of 3D scanning to obtain custom virtual bodies (39) along with the use of 3D virtualization programs to aid in pattern making (40). Such virtual garments could fast-track prototyping and reduce preproduction costs. Follow up efforts with pain patients could further elucidate fabric behavior properties as well as guide the customization of parametric models to fit specific pain conditions.

## Discussion

The perspective on the current scientific literature of sartorial accommodations for pain patients exposes multiple crucial needs that need to be met. First, the need for specialized garments targeted for select pain populations: this can be challenging since a garment designed to help fibromyalgia patients may be completely inefficient for those with headaches, complex regional pain syndrome, back pain, etc. Second, the need to listen to the pain patient: most patients are not satisfied with therapeutic garments/footwear (41), which in itself may decrease both treatment efficacy and compliance. For example, therapeutic footwear had a negative impact on female patients with RA (42). Furthermore, there is a clear bidirectional modulation between pain and clothing. Being unable to dress comfortably exacerbates pain conditions and patient well-being (43). Patient-guided design could therefore improve compliance since garments and footwear are not simply functional, but tools of self-expression. Third, the need to consider custom-designed as opposed to ready-made apparel: cost considerations are often the limiting factor here, but patient heterogeneity necessitates the design of personalized garments that may better address the patient's needs, even within the same pain syndrome. Advances in technology will facilitate such efforts; in a recent case study, a simple finite element model was used and found to possess an acceptable degree of accuracy when creating orthoses for a patient with diabetes (44). Should therapeutic apparel become mainstream treatments, it is possible to envision them grouped with other treatment expenses.

The proposed perspective also highlights the limited efficacy of garments and footwear currently in use. This seemingly low efficacy in clinical trials could be due to many factors including efficacious placebo response, (lack of) patient compliance, patient heterogeneity, comorbid conditions, large variations in study methodology, etc. It could also reflect the limited availability of options regarding adaptive clothing. It is entirely possible that the interdisciplinary nature of such an approach leaves adaptive garments in no-man's land between the distinct worlds of fashion and medicine. Finally, rigorous mechanistic studies are needed prior to affirming the therapeutic value of adaptive garments and footwear. To date, there are no such studies indicating the exact physiological mechanisms, partly since we are unable to use preclinical models to test such interventions. For instance, efficacy could be due to changes in cutaneous stimulation or secondary to improvement in overall well-being, indicating the involvement of supra-spinal mechanisms. Such mechanisms have been described for other complementary and alternative therapies for pain, such as exercise and meditation, as described in a recent review (45).

The global apparel industry is predicted to reach an approximate worth of 2.23 trillion dollars by the year 2025 (46). Out of this colossal industry, only a negligible percentage is targeted toward the millions of pain sufferers worldwide. It is time for the pain patient to be fairly represented in the customer base, an initiative that would open entirely novel alterative and complementary pain therapies for pain prevention and treatment.

## Data Availability Statement

The original contributions presented in the study are included in the article/supplementary material, further inquiries can be directed to the corresponding author/s.

## Author Contributions

MT conceived the original idea and wrote the perspective with support from JG. All authors contributed to the article and approved the submitted version.

## Funding

MT was supported by NIH grant 1 SC2 GM135114-01.

## Conflict of Interest

The authors declare that the research was conducted in the absence of any commercial or financial relationships that could be construed as a potential conflict of interest.

## Publisher's Note

All claims expressed in this article are solely those of the authors and do not necessarily represent those of their affiliated organizations, or those of the publisher, the editors and the reviewers. Any product that may be evaluated in this article, or claim that may be made by its manufacturer, is not guaranteed or endorsed by the publisher.
